# Distribution of the Duffy genotypes in Malaysian Borneo and its relation to *Plasmodium knowlesi* malaria susceptibility

**DOI:** 10.1371/journal.pone.0222681

**Published:** 2019-09-19

**Authors:** Jeremy Ryan de Silva, Amirah Amir, Yee Ling Lau, Choo-Huck Ooi, Mun Yik Fong

**Affiliations:** 1 Department of Parasitology, Faculty of Medicine, University of Malaya, Kuala Lumpur, Malaysia; 2 Sarawak State Health Department, Jalan Diplomatik, Off Jalan Bako, Kuching, Sarawak, Malaysia; Agency for Science, Technology and Research - Singapore Immunology Network, SINGAPORE

## Abstract

The Duffy blood group plays a key role in *Plasmodium knowlesi* and *Plasmodium vivax* invasion into human erythrocytes. The geographical distribution of the Duffy alleles differs between regions with the FY*A allele having high frequencies in many Asian populations, the FY*B allele is found predominately in European populations and the FY*B^*es*^ allele found predominantly in African regions. A previous study in Peninsular Malaysia indicated high homogeneity of the dominant FY*A/FY*A genotype. However, the distribution of the Duffy genotypes in Malaysian Borneo is currently unknown. In the present study, the distribution of Duffy blood group genotypes and allelic frequencies among *P*. *knowlesi* infected patients as well as healthy individuals in Malaysian Borneo were determined. A total of 79 *P*. *knowlesi* patient blood samples and 76 healthy donor samples were genotyped using allele specific polymerase chain reaction (ASP-PCR). Subsequently a *P*. *knowlesi* invasion assay was carried out on FY*AB/ FY*A and FY*A/ FY*A Duffy genotype blood to investigate if either genotype conferred increased susceptibility to *P*. *knowlesi* invasion. Our results show almost equal distribution between the homozygous FY*A/FY*A and heterozygous FY*A/FY*B genotypes. This is in stark contrast to the Duffy distribution in Peninsular Malaysia and the surrounding Southeast Asian region which is dominantly FY*A/FY*A. The mean percent invasion of FY*A/FY*A and FY*A/FY*B blood was not significantly different indicating that neither blood group confers increased susceptibility to *P*. *knowlesi* invasion.

## Introduction

*Plasmodium knowlesi* is a monkey malaria parasite that infects humans and causes significant mortality and morbidity in endemic areas. This parasite is transmitted via the bite of the *Anopheles* mosquito of the Leucosphyrus group. Following the discovery of a large number of human *P*. *knowlesi* infections in Sarawak, Borneo Malaysia in 2004 [1], similar infections have been reported in all countries in Southeast Asia, the China-Myanmar border and the Andaman and Nicobar islands in the Indian Ocean [2–5]. In Malaysia, *P*. *knowlesi* has replaced *Plasmodium vivax* as the main cause of human malaria infections in the country with 3164 cases in 2017 most of which occur in Malaysian Borneo [6].

Malaria as an infectious disease is generally recognized as one of the factors that contributes to variability in the human Duffy blood group system. The Duffy blood group was first described by Cutbush in 1950 through the discovery of an antibody in a multitransfused hemophiliac patient [7]. This antibody was reactive with an antigen, denoted as Fy^a^. An allelic variant of the antigen, Fy^b^, was identified a year later [8]. The Duffy antigens occur on the surface of erythrocytes and are encoded by the *DARC* (Duffy antigen receptor for chemokines) gene located on the long arm of chromosome 1 (1.q22-1.q23) [9]. This gene has two major codominant alleles, FY*A and FY*B which differ from each other by a missense mutation in the major cDNA transcript (G125A>Gly42Asp) [10]. A third allele, FY*B^*es*^, is a result of a substitution at the GATA box motif of the FY*B promoter (-33 T>C) [11]. This mutation results in the disruption at the binding site of the GATA-1 erythroid transcription factor which in turn results in the loss of FY expression in the erythroid lineage. These alleles confer the common Duffy phenotypes Fy(a+b+), Fy(a+b-), Fy(a-b+) and Fy(a-b-).

The geographical distribution of the Duffy alleles differ between regions with the FY*A allele having high frequencies in many Asian populations, while the FY*B allele is found predominately in European populations. This differs in most of the African regions where the FY*B^*es*^ is the dominant allele [12]. The DARC is a receptor for chemokines and plays a crucial role in the regulation of circulating chemokine levels [13]. Studies have associated Duffy polymorphisms with resistance or susceptibility to disease [14] and the absence of the Duffy blood group to lower neutrophil count [15]. The DARC also plays a critical function for the entry of *P*. *vivax* and *P*. *knowlesi* into human erythrocytes. Erythrocytes of the Fy(a-b-) phenotype are found to be refractory to invasion by these merozoites [16–18].

Our previous study outlined the Duffy allele frequencies, genotypes and phenotypes of *P*. *knowlesi* infected patients and healthy donors from Peninsular Malaysia, in which a dominant FY*A allelic and FY*A/FY*A genotypic distribution (>89%) was observed [19]. However, to date there is no data on the distribution of the Duffy blood group in Malaysian Borneo. Interestingly, it has been observed that the Malaysian Borneo has more cases of *P*. knowlesi infection and more occurrences of hyperparasitemia or severe cases compared to Peninsular Malaysia [20–22]. The reason for these increased occurrences are currently unknown. Thus, the current study aims to identify the frequency distribution of the Duffy alleles and genotypes in Malaysian Borneo and determine if a particular Duffy genotype confers increased susceptibility to *P*. *knowlesi* infection.

## Materials and methods

### Blood samples and sample collection

*P*. *knowlesi* patient blood samples (n = 79) were collected from clinics in Malaysian Borneo states including the Sabah and Sarawak states from 2012 to 2018. Symptomatic patient blood samples were confirmed for *P*. *knowlesi* infection via microscopic examination of thick and thin smears and PCR based on the *Plasmodium* small subunit ribosomal RNA genes [1, 23]. Blood samples from a control group of healthy donors (n = 76) were also collected from Malaysian Borneo and included in the study. These healthy donors had been diagnosed as malaria negative by PCR and had no previous malarial infections. A total of 120 Sarawak and 35 Sabah samples were obtained via convenience sampling for the purposes of this study. To minimize participation bias, only patients that were symptomatic for malaria were invited to participate in this study. Samples were collected from various districts hospitals in the Sabah and Sarawak state of Malaysia instead of a single sampling site to ensure that the samples are representative of a large geographic region of Malaysian Borneo. Furthermore, it should be noted that Malaysian Borneo is ethnically diverse which includes native indigenous groups, Chinese, and Malays. Thus, for the purpose of this study, only the indigenous native blood samples were used to ensure that the results of the genotyping study were a true representation of the Duffy distribution in the native Malaysian Borneo population.

Ethical approval for this study was obtained from the University of Malaya Medical Centre Ethic Committee (MEC Ref. No. 817.18) and informed written or verbal consent from the donor or the next of kin was obtained for use of these samples in diagnosis. Verbal consent was used to obtain patient consent if the participant was found to be illiterate and unable to give written consent. This consent procedure was approved by the University of Malaya Medical Centre Ethic Committee.

### Genomic DNA extraction and allele-specific PCR (ASP-PCR)

Genomic DNA was extracted from blood samples using a commercial blood extraction kit according to the manufacturer’s protocol (QIAGEN, Hilden, Germany). Allele specific-PCR was used to determine the Duffy blood group genotypes based on four sets of primers as previously described [19]. Briefly, each genomic DNA sample was subjected to three different PCR reactions to amplify the FY*A, FY*B and FY*B^*es*^ gene respectively using a combination of the four primers mentioned. Two of these PCR reactions used the *FY* forward primer paired with either the FY*A or FY*B specific reverse primer. The third reaction used a combination of a forward primer that annealed to the mutated promoter region of the FY*B^*es*^ and the FY*B specific reverse primer. The expected size for the PCR product was 713 base pairs in length.

PCR conditions that were previously optimized were used to amplify the Duffy alleles [19]. The PCR reaction was set up in a 20 μl reaction with 0.5 μg of genomic DNA, 0.4 μM of forward and reverse primer, 0.2 μmM of dNTP, 2 mM MgCl_2_ and 1 unit of Taq DNA polymerase in the appropriate buffer (Promega, WI, U.S.A.). PCR conditions were initiated with an initial denaturation of one cycle at 94 °C for 2 minutes followed by 30 cycles of 30 seconds at 94 °C, 1 minute at 60 °C for annealing and 1 minute at 72 °C performed in a Biorad MyCycler thermal cycler (Biorad, CA, U.S.A.). All PCR reactions were terminated after a 10 minute extension at 72 °C and PCR products were analyzed by gel electrophoresis on a 2% agarose gel stained with SYBR Safe DNA gel stain (Invitrogen, OR, U.S.A.).

### *P*. *knowlesi* culture

Human-adapted *P*. *knowlesi* A1-H1 strain (a kind gift from Robert W. Moon, London School of Hygiene and Tropical Medicine, London, UK) was maintained with fresh human erythrocytes in RPMI-1640- based medium (Invitrogen Life Technologies, NY, U.S.A.) and *P*. *knowlesi* schizonts were purified as previously described [24].

### *P*. *knowlesi* invasion assay

Purified schizont preparations were mixed separately with FY*AB/ FY*A and FY*A/ FY*A Duffy genotype blood such that the starting schizont parasitemia was no more than 12%. The respective mixture was then diluted to 4% haematocrit using complete RPMI-1640 media. Aliquots of 100μl of each mixture were then distributed into 96-well plates and gassed with 90% N_2_, 5% O_2_, and 5% CO_2_. The cultures were then incubated at 37°C for an average of 15 hours, depending on the stage of parasite maturation to allow for parasite re-invasion to occur. Technical replicates were prepared for both the FY*AB/ FY*A and FY*A/ FY*A blood cultures. Thin blood smears were made at the end of the incubation period when newly invaded ring-stage parasites were found. The number of rings/trophozoite in 4000 erythrocytes was counted to determine the mean invasion percentage.

### Statistical analysis

Gene frequencies were calculated by the gene-counting method. Fisher’s exact test was used to evaluate the significance of the genotypic and allelic variables and to assess the independence among the proportions of Duffy genotype alleles in *P*. *knowlesi* infected patients and blood donors. The independent non-parametric Mann-Whitney test was used to determine the differences between the means of the FY*AB/ FY*A and FY*A/ FY*A in the *P*. *knowlesi* invasion assay with 95% CI (GraphPad Prism Ver. 5.01). A two-tailed P-value equal to or less than 0.05 was considered statistically significant.

## Results

A total of 155 blood samples were genotyped, 76 of which were from healthy donors and 79 from *P*. *knowlesi* infected patients. The proportion of Duffy genotype frequencies between healthy donors and *P*. *knowlesi* positive samples is summarized in Table 1. The results show almost equal distribution between the homozygous FY*A/FY*A and heterozygous FY*A/FY*B genotypes. Healthy donors have 45% and 55% genotype distribution and *P*. *knowlesi* positive samples with 53% and 47% distribution respectively. This is reflected in the proportion of predicted phenotypes for both groups, with the Fy (a+b-) and Fy (a+b+) phenotypes occurring at similar frequencies of 49% and 50% respectively (Table 2). There were no significant differences between the frequencies of either the FY*A/FY*A or FY*A/FY*B genotypes between healthy donors or *P*. *knowlesi* patient samples. The homozygous FY*B/FY*B genotype and corresponding Fy (a-b+) phenotype was only observed in one individual from the healthy donor group. No Duffy negative genotype (FY*B^*ES*^/FY*B^*ES*^) or Fy (a-b-) phenotype was detected.

**Table 1:** Comparison of genotypic frequencies between blood donors and *P*. *knowlesi* infected patients.

Genotypes	Donors (n = 76)	Patients *P*. *knowlesi* (n = 79)	P-value
FY*A/FY*B	41 (55%)	37 (47%)	0.423
FY*A/FY*A	34 (45%)	42 (53%)	0.336
FY*A/FY*B^***ES***^	0	0	
FY*B/FY*B	1(1%)	0	0.490
FY*B/FY*B^***ES***^	0	0	
FY*B^***ES***^/FY*B^***ES***^	0	0	

**Table 2:** Proportion of predicted phenotypes of blood donors and *P*. *knowlesi* infected patients.

Predicted phenotype	No. of individuals
**Fy (a+b+)**	78 (50%)
**Fy (a+b-)**	76 (49%)
**Fy (a-b+)**	1 (1%)
**Fy (a-b-)**	0

As for allelic distribution (Table 3), the FY*A was found to be the predominant allele in both groups, with a frequency of 0.717 in healthy donors and 0.766 in *P*. *knowlesi* patient samples. The FY*B allele had allelic frequency of 0.283 and 0.234 in healthy donors and *P*. *knowlesi* patients respectively. The FY*B^*ES*^ allele was not detected in either group. The distribution of the FY*A and FY*B alleles was not significantly different (P = 0.728, P = 0.531) between the healthy donor and *P*. *knowlesi* samples.

**Table 3:** Comparison of allelic frequencies between blood donors and *P*. *knowlesi* infected patients.

Alleles	Donors (n = 76)	Patients *P*. *knowlesi* (n = 79)	P-value
FY*A	0.717 (72%)	0.766 (77%)	0.728
FY*B	0.283 (28%)	0.234 (23%)	0.531
FY*B^***ES***^	0	0	

**Fig 1:**
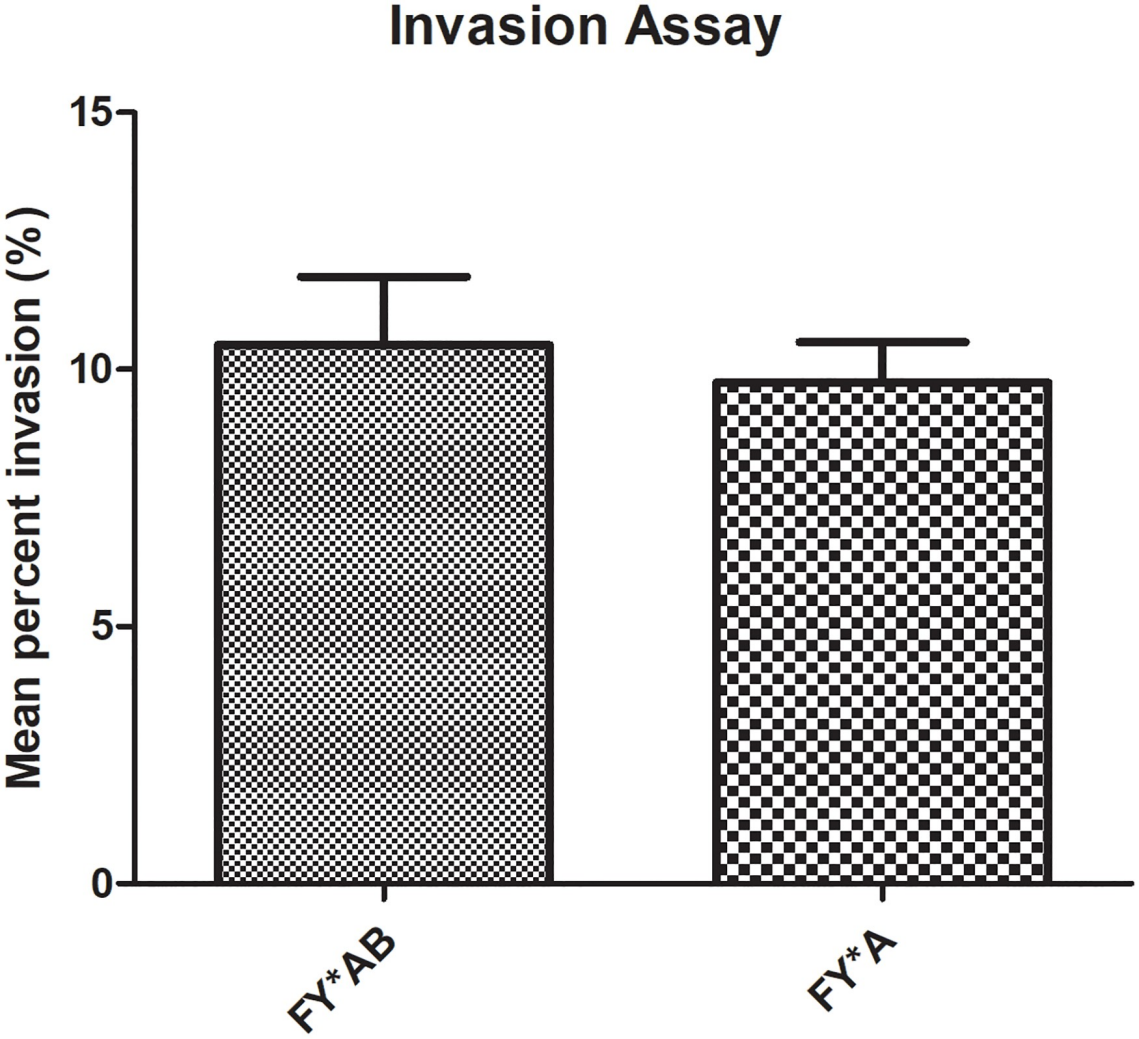
Mean percent invasion of *P*. *knowlesi* in FY*AB/ FY*A and FY*A/ FY*A Duffy blood groups. Data shown was the mean percent invasion with standard deviation in each group. The difference between FY*A/FY*A or FY*A/FY*B genotypes was not significantly different. *P<0.05.

To further study the susceptibility of the two predominant Duffy genotypes (FY*AB/ FY*A and FY*A/ FY*A) to *P*. *knowlesi* invasion, an *in vitro* invasion assay was conducted. The mean percent invasion for FY*AB/ FY*A and FY*A/ FY*A were 10.48% and 9.753% respectively (Table 4). Statistical analysis shows that there was no significant difference between the mean percent invasion for both Duffy blood genotypes (Fig 1) indicating that neither blood group has increased susceptibility to *P*. *knowlesi* infection.

**Table 4:** Comparison of invasion percentage between the Duffy FY*AB and FY*A blood group.

	FY*AB/ FY*A percent invasion (%) ± SD	FY*A/ FY*A percent invasion (%) ± SD	P-value
Replicate 1	11.57	9.45	
Replicate 2	10.86	10.64	
Replicate 3	9.00	9.17	
Mean percent invasion	10.48±1.33	9.75±0.78	0.700

## Discussion

The Duffy blood group system has been widely used for population differentiation and ethnic composition studies. It has also been a popular chromosomal marker for evaluating the impact of natural selection in different geographical locations [25, 26]. The Duffy blood group also has an important role in transfusion medicine [27, 28], and invasion of *P*. *vivax* and *P*. *knowlesi* into erythrocytes [16, 17]. In the present study we looked at the distribution of the Duffy blood group in Malaysian Borneo and determined its possible role in susceptibility to *P*. *knowlesi* infection.

The Duffy blood group distribution is well studied and characterized, with the FY*A allele being the most abundant globally [12]. This allele clusters primarily in the Asian continent, Australia and eastern parts of Russia. The FY*B allele on the other hand is found to cluster primarily in the European continent and in pockets of the Americas and its frequency decreases eastwards towards the Asia. The FY*B^*ES*^ allele on the other hand is found to be primarily distributed in the African region where it has reached fixation across parts of west, central, and east Africa. The allelic frequencies of 30 countries across Africa were found to be >90% FY*B^*ES*^ [12].

Our previous study on the Duffy blood group in Peninsular Malaysia was in agreement with this trend, indicating an overwhelmingly FY*A dominant distribution with allelic frequencies of 0.937 (94%) and FY*A/FY*A being the dominant genotype at 89.2% [19]. This allelic and genotypic distribution was similar to those of neighbouring countries such as Thailand [29, 30], Indonesia (Bugis, Toraja, and Irian Jaya area) [31], Philippines [32], Taiwan [32], and China [33, 34]. With our previous study on the Duffy genotypes in Peninsular Malaysia in mind, we sought to determine if similar distributions were observed in Malaysian Borneo or if the distributions would differ due to the geographic separation of the Borneo island. To that end we collected new samples from a cohort of Malaysian Borneo *P*. *knowlesi* infected patients and healthy donors. We found that the data on the Duffy genotypes in Malaysian Borneo markedly contrast with that of the data of the Duffy genotypes in Peninsular Malaysia from our previous study and those of the neighbouring regions, with the almost similar distribution of the FY*AB/ FY*A and FY*A/ FY*A at 50% and 49% respectively. Furthermore, the allelic distributions were 0.724 (74.2%) and 0.252 (25.2%) for the FY*A and FY*B allele respectively. This is particularly interesting as the countries that geographically surround Malaysian Borneo i.e. Peninsular Malaysia, Indonesia, and the Philippines are predominantly of the FY*A/FY*A genotype (>80%). This also differs greatly from the global distribution of the Duffy genotypes in which mainland Asian and Southeast Asian regions show FY*A allele dominance [12, 35].

Admixture of FY*A and FY*B in a population may be due to movement of migrants globally over time as can be seen in the evolution of Duffy genotypes in North America and the Americas with migrations from the European continent and early migration from Asian population [35]. Similarly, the admixture of the FY*B^*ES*^ allele seen in Brazil and other regions in the Americas were noted in individuals with African ancestry [36]. This however does not seem to be the case in our findings as the surrounding regions around Malaysian Borneo are of a predominantly FY*A/FY*A genotype thus population movement would not explain the high incidence of FY*A/FY*B genotypes. Furthermore, the target population sampled for this study was of native indigenous Borneo ancestry without any admixture of other ethnicities indicating that this distribution is unlikely to be due to admixture with other populations in or around the region. There is currently no studies available on the Duffy allele distribution in Brunei or the Kalimantan region of Indonesia, thus it is unknown if the Duffy allele distribution of the entire Borneo island is similarly distributed.

Following the discovery of this pocket of FY*A/FY*AB in a predominantly FY*A/FY*A region, we decided to investigate if either genotype confers an increased susceptibility to *P*. *knowlesi* infection. Previous studies have shown that the *P*. *vivax* Duffy Binding Protein-II (PvDBPII), a receptor that facilitates invasion, has significantly lower binding to the Fy^a^ antigen compared to Fy^b^. Furthermore, King *et al* found that Fy^a^ was associated with protection but Fy^b^ was associated with increased infection and disease [35]. A study by Miller *et al* suggested that *P*. *knowlesi* had a lower efficiency in infecting human Fy^a+b-^ compared to Fy^a-b+^ [17]. Similarly, *in vitro* invasion studies of *P*. *knowlesi* have shown a reduced efficiency of invasion for Fy (a+b-) erythrocytes compared to Fy(a-b+) erythrocytes [37]. These prior studies indicate that the FY*A allele confers some resistance to infection and conversely FY*B is associated with increased susceptibility to *P*. *knowlesi* invasion. A previous study on *P*. *knowlesi* Duffy Binding Protein II (PkDBPαII) found that this protein showed different binding activity between Malaysian Peninsular and Borneo erythrocytes [38]. Further studies showed that the PkDBPαII protein had a higher binding activity to Fy (a+b+) compared to Fy (a+b-) erythrocytes [39].

However, our analysis of the mean percent invasion of FY*AB/ FY*A and FY*A/ FY*A erythrocytes showed no significant difference in invasion rates between these two genotypes. Although a prior study by Miller *et al* [17] observed a marked reduction in *P*. *knowlesi* invasion of Fy (a+b-) erythrocytes compared to Fy (a-b+) it should be noted that this was done via an invasion inhibition assay using Duffy positive erythrocytes coated with anti-Fy^a^ and anti- Fy^b^ antiserum and may not accurately mimic natural *P*. *knowlesi* invasion into different Duffy genotype erythrocytes. Indeed, in the same paper the authors also carried out an invasion assay using different duffy genotype erythrocytes and noted that the invasion rates for three Duffy positive genotypes, Fy (a+b-+), Fy (a-b+), and Fy (a+b+) were similar which is consistent with our findings. Our previous study also found that the binding activity of PkDBPαII was higher in Fy (a+b+) compared to Fy (a+b-) erythrocytes. However, binding efficiency and rosette formation are not currently linked to *P*. *knowlesi* invasion into human RBC’s, thus binding efficiency may not always be concordant with *P*. *knowlesi* invasion rates.

In *P*. *vivax* endemic areas such as Iran [40], where the frequency of Fy (a+b-) was 11% higher in vivax patients compared to healthy donors; India [41], where the prevalence of Duffy Fy (a+b-) was 83.3% in malaria patients compared to (59.0%) healthy participants; and Brazil [42], where Fy (a+b-) was found to be significantly higher in *P*. *vivax* patients (34.93%) compared to healthy donors (27.27%); it was noted that the Duffy Fy (a+b-) carried higher susceptibility for *P*. *vivax* infection. Thus, the question arose if the Duffy genotypes played a similar role in increasing susceptibility to *P*. *knowlesi* infection. The results of our genotyping however show that there is no significant difference in the distribution of FY*AB/ FY*A and FY*A/ FY*A between *P*. *knowlesi* infected patient samples and healthy donors. This indicates that neither the FY*AB/ FY*A nor the FY*A/ FY*A genotype confers any differences in susceptibility to *P*, *knowlesi* infection. We further studied this *in vitro* via an invasion assay and the results of the assay were concordant with our genotyping results where it was observed that there was no significant difference between the mean percent invasion of FY*AB/ FY*A and FY*A/ FY*A erythrocytes. Thus, unlike *P*. *vivax*, *P*. *knowlesi* infects erythrocytes indiscriminately regardless of FY*A or FY*B genotype.

Conducting an invasion inhibition assay using anti- Fy^a^ and anti- Fy^b^ antibodies to block a combination of these specific antigens on human erythrocytes would allow for a more robust investigation of *P*. *knowlesi* invasion rates between different Duffy genotypes. However, this does come at the expense of observing natural *P*. *knowlesi* invasion rates in invasion assay using different Duffy genotype erythrocytes. Ideally, conducting invasion assays using different Duffy genotype erythrocytes in comparison with invasion inhibition assays using anti- Fy^a^ and anti- Fy^b^ antibodies would allow for a more comprehensive analysis of the *P*. *knowlesi* invasion rates between the different Duffy genotypes and is a future study that merits investigation. Another limitation of the study is the lack of complete sociodemographic data, thus we chose to omit this data from the study. Ideally, our future studies on Borneo genotyping will include complete patient data to study the influence of sociodemographic factors on knowlesi susceptibility.

### Conclusion

This is a first report on the Duffy blood group distribution in Malaysian Borneo and the first effort at studying the difference in *P*. *knowlesi* invasion between Duffy Fy (a+b+) and Fy (a+b-) erythrocytes. Our results revealed an interesting cluster of the FY*A/FY*B genotype in Malaysian Borneo which is in stark contrast to surrounding regions and global expected Duffy blood group distributions. Our results have also shown that there is no significant difference in susceptibility to *P*. *knowlesi* infection between the FY*A/FY*A and FY*A/FY*B genotype. Further studies into the Duffy distribution in the Kalimantan region of Indonesia and in Brunei would help define if this FY*A/FY*B distribution is observed throughout the entire Borneo island thus providing merit for further investigation into the cause of this unique admixture of Duffy alleles in this geographic region.

## References

[1] Singh B, Kim Sung L, Matusop A, Radhakrishnan A, Shamsul SS, Cox-Singh J, et al A large focus of naturally acquired Plasmodium knowlesi infections in human beings. Lancet. 2004;363(9414):1017–24. Epub 2004/03/31. 10.1016/S0140-6736(04)15836-4 . 15051281

[2] Moyes CL, Henry AJ, Golding N, Huang Z, Singh B, Baird JK, et al Defining the geographical range of the Plasmodium knowlesi reservoir. PLoS Negl Trop Dis. 2014;8(3):e2780 Epub 2014/03/29. 10.1371/journal.pntd.0002780 . 24676231 PMC3967999

[3] Jongwutiwes S, Putaporntip C, Iwasaki T, Sata T, Kanbara H. Naturally acquired Plasmodium knowlesi malaria in human, Thailand. Emerg Infect Dis. 2004;10(12):2211–3. Epub 2005/01/25. 10.3201/eid1012.040293 . 15663864 PMC3323387

[4] Ng OT, Ooi EE, Lee CC, Lee PJ, Ng LC, Pei SW, et al Naturally acquired human Plasmodium knowlesi infection, Singapore. Emerg Infect Dis. 2008;14(5):814–6. Epub 2008/04/29. 10.3201/eid1405.070863 . 18439370 PMC2600232

[5] Zhu HM, Li J, Zheng H. [Human natural infection of Plasmodium knowlesi]. Zhongguo Ji Sheng Chong Xue Yu Ji Sheng Chong Bing Za Zhi. 2006;24(1):70–1. Epub 2006/07/27. . 16866152

[6] Malaysia Ministry of Health. Zoonotic Malaria And The Prevention Program In Malaysia. 2018. http://www.moh.gov.my/index.php/database_stores/store_view_page/21/1087.

[7] Cutbush M, Mollison PL. The Duffy blood group system. Heredity (Edinb). 1950;4(3):383–9. Epub 1950/12/01. 10.1038/hdy.1950.31 . 14802995

[8] Ikin EW, Mourant AE, Pettenkofer HJ, Blumenthal G. Discovery of the expected haemagglutinin, anti-Fyb. Nature. 1951;168(4288):1077–8. Epub 1951/12/22. 10.1038/1681077b0 . 14910641

[9] Mathew S, Chaudhuri A, Murty VV, Pogo AO. Confirmation of Duffy blood group antigen locus (FY) at 1q22—>q23 by fluorescence in situ hybridization. Cytogenet Cell Genet. 1994;67(1):68 Epub 1994/01/01. 10.1159/000133801 . 8187556

[10] Chaudhuri A, Polyakova J, Zbrzezna V, Pogo AO. The coding sequence of Duffy blood group gene in humans and simians: restriction fragment length polymorphism, antibody and malarial parasite specificities, and expression in nonerythroid tissues in Duffy-negative individuals. Blood. 1995;85(3):615–21. Epub 1995/02/01. . 7833466

[11] Iwamoto S, Li J, Sugimoto N, Okuda H, Kajii E. Characterization of the Duffy gene promoter: evidence for tissue-specific abolishment of expression in Fy(a-b-) of black individuals. Biochem Biophys Res Commun. 1996;222(3):852–9. Epub 1996/05/24. 10.1006/bbrc.1996.0833 . 8651934

[12] Howes RE, Patil AP, Piel FB, Nyangiri OA, Kabaria CW, Gething PW, et al The global distribution of the Duffy blood group. Nat Commun. 2011;2:266 Epub 2011/04/07. 10.1038/ncomms1265 . 21468018 PMC3074097

[13] Lee JS, Frevert CW, Wurfel MM, Peiper SC, Wong VA, Ballman KK, et al Duffy antigen facilitates movement of chemokine across the endothelium in vitro and promotes neutrophil transmigration in vitro and in vivo. J Immunol. 2003;170(10):5244–51. Epub 2003/05/08. 10.4049/jimmunol.170.10.5244 . 12734373 PMC4357319

[14] Anstee DJ. The relationship between blood groups and disease. Blood. 2010;115(23):4635–43. Epub 2010/03/24. 10.1182/blood-2010-01-261859 . 20308598

[15] Reich D, Nalls MA, Kao WH, Akylbekova EL, Tandon A, Patterson N, et al Reduced neutrophil count in people of African descent is due to a regulatory variant in the Duffy antigen receptor for chemokines gene. PLoS Genet. 2009;5(1):e1000360 Epub 2009/01/31. 10.1371/journal.pgen.1000360 . 19180233 PMC2628742

[16] Miller LH, Mason SJ, Clyde DF, McGinniss MH. The resistance factor to Plasmodium vivax in blacks. The Duffy-blood-group genotype, FyFy. N Engl J Med. 1976;295(6):302–4. Epub 1976/08/05. 10.1056/NEJM197608052950602 . 778616

[17] Miller LH, Mason SJ, Dvorak JA, McGinniss MH, Rothman IK. Erythrocyte receptors for (Plasmodium knowlesi) malaria: Duffy blood group determinants. Science. 1975;189(4202):561–3. Epub 1975/08/15. 10.1126/science.1145213 . 1145213

[18] Barnwell JW, Nichols ME, Rubinstein P. In vitro evaluation of the role of the Duffy blood group in erythrocyte invasion by Plasmodium vivax. J Exp Med. 1989;169(5):1795–802. Epub 1989/05/01. 10.1084/jem.169.5.1795 . 2469769 PMC2189319

[19] De Silva JR, Lau YL, Fong MY. Genotyping of the Duffy blood group among Plasmodium knowlesi-infected patients in Malaysia. PLoS One. 2014;9(9):e108951 Epub 2014/10/01. 10.1371/journal.pone.0108951 . 25268233 PMC4182577

[20] Barber BE, William T, Grigg MJ, Menon J, Auburn S, Marfurt J, et al A prospective comparative study of knowlesi, falciparum, and vivax malaria in Sabah, Malaysia: high proportion with severe disease from Plasmodium knowlesi and Plasmodium vivax but no mortality with early referral and artesunate therapy. Clin Infect Dis. 2013;56(3):383–97. Epub 2012/10/23. 10.1093/cid/cis902 . 23087389

[21] William T, Menon J, Rajahram G, Chan L, Ma G, Donaldson S, et al Severe Plasmodium knowlesi malaria in a tertiary care hospital, Sabah, Malaysia. Emerg Infect Dis. 2011;17(7):1248–55. Epub 2011/07/19. 10.3201/eid1707.101017 . 21762579 PMC3381373

[22] Singh B, Daneshvar C. Plasmodium knowlesi malaria in Malaysia. Med J Malaysia. 2010;65(3):166–72. Epub 2011/09/24. . 21939162

[23] Imwong M, Tanomsing N, Pukrittayakamee S, Day NP, White NJ, Snounou G. Spurious amplification of a Plasmodium vivax small-subunit RNA gene by use of primers currently used to detect P. knowlesi. J Clin Microbiol. 2009;47(12):4173–5. Epub 2009/10/09. 10.1128/JCM.00811-09 . 19812279 PMC2786678

[24] Amir A, Russell B, Liew JW, Moon RW, Fong MY, Vythilingam I, et al Invasion characteristics of a Plasmodium knowlesi line newly isolated from a human. Sci Rep. 2016;6:24623 Epub 2016/04/22. 10.1038/srep24623 . 27097521 PMC4838912

[25] Hamblin MT, Di Rienzo A. Detection of the signature of natural selection in humans: evidence from the Duffy blood group locus. Am J Hum Genet. 2000;66(5):1669–79. Epub 2000/04/14. 10.1086/302879 . 10762551 PMC1378024

[26] Hamblin MT, Thompson EE, Di Rienzo A. Complex signatures of natural selection at the Duffy blood group locus. Am J Hum Genet. 2002;70(2):369–83. Epub 2001/12/26. 10.1086/338628 . 11753822 PMC419988

[27] Noizat-Pirenne F. Relevance of blood groups in transfusion of sickle cell disease patients. C R Biol. 2013;336(3):152–8. Epub 2013/05/07. 10.1016/j.crvi.2012.09.011 . 23643398

[28] Babinszki A, Berkowitz RL. Haemolytic disease of the newborn caused by anti-c, anti-E and anti-Fya antibodies: report of five cases. Prenat Diagn. 1999;19(6):533–6. Epub 1999/07/23. 10.1002/(sici)1097-0223(199906)19:6<533::aid-pd570>3.0.co;2-5 . 10416968

[29] Nathalang O, Intharanut K, Siriphanthong K, Nathalang S, Kupatawintu P. Duffy blood group genotyping in Thai blood donors. Ann Lab Med. 2015;35(6):618–23. Epub 2015/09/12. 10.3343/alm.2015.35.6.618 . 26354350 PMC4579106

[30] Nathalang O, Kuvanont S, Punyaprasiddhi P, Tasaniyanonda C, Sriphaisal T. A preliminary study of the distribution of blood group systems in Thai blood donors determined by the gel test. Southeast Asian J Trop Med Public Health. 2001;32(1):204–7. Epub 2001/08/04. . 11485086

[31] Shimizu Y, Ao H, Soemantri A, Tiwawech D, Settheetham-Ishida W, Kayame OW, et al Sero- and molecular typing of Duffy blood group in Southeast Asians and Oceanians. Hum Biol. 2000;72(3):511–8. Epub 2000/07/08. . 10885196

[32] Peng CT, Tsai CH, Lee HH, Lin CL, Wang NM, Chang JG. Molecular analysis of Duffy, Yt and Colton blood groups in Taiwanese, Filipinos and Thais. Kaohsiung J Med Sci. 2000;16(2):63–7. Epub 2000/05/19. . 10816988

[33] Liu Z, Zeng R, Chen Q, Li M, Shi GY, Wei P, et al Genotyping for Kidd, Kell, Duffy, Scianna, and RHCE blood group antigens polymorphisms in Jiangsu Chinese Han. Chin Med J (Engl). 2012;125(6):1076–81. Epub 2012/05/23. . 22613534

[34] Zhou S, Liu M, An W, Liang X, Yu W, Piao F. A New Method for Analyzing the Duffy Blood Group Genotype by TaqMan Minor Groove Binding Probes. J Clin Lab Anal. 2015;29(3):203–7. Epub 2014/05/07. 10.1002/jcla.21751 . 24798509 PMC6807056

[35] King CL, Adams JH, Xianli J, Grimberg BT, McHenry AM, Greenberg LJ, et al Fy(a)/Fy(b) antigen polymorphism in human erythrocyte Duffy antigen affects susceptibility to Plasmodium vivax malaria. Proc Natl Acad Sci U S A. 2011;108(50):20113–8. Epub 2011/11/30. 10.1073/pnas.1109621108 . 22123959 PMC3250126

[36] Martins ML, da Silva AR, Santos HC, Alves MT, Schmidt LC, Vertchenko SB, et al Duffy blood group system: New genotyping method and distribution in a Brazilian extra-Amazonian population. Mol Cell Probes. 2017;35:20–6. Epub 2017/06/08. 10.1016/j.mcp.2017.06.001 . 28587995

[37] Mason SJ, Miller LH, Shiroishi T, Dvorak JA, McGinniss MH. The Duffy blood group determinants: their role in the susceptibility of human and animal erythrocytes to Plasmodium knowlesi malaria. Br J Haematol. 1977;36(3):327–35. Epub 1977/07/01. 70210. 10.1111/j.1365-2141.1977.tb00656.x 70210

[38] Lim KL, Amir A, Lau YL, Fong MY. The Duffy binding protein (PkDBPalphaII) of Plasmodium knowlesi from Peninsular Malaysia and Malaysian Borneo show different binding activity level to human erythrocytes. Malar J. 2017;16(1):331 Epub 2017/08/13. 10.1186/s12936-017-1984-8 . 28800732 PMC5553923

[39] Fong MY, Cheong FW, Lau YL. Erythrocyte-binding assays reveal higher binding of Plasmodium knowlesi Duffy binding protein to human Fy(a+/b+) erythrocytes than to Fy(a+/b-) erythrocytes. Parasit Vectors. 2018;11(1):527 Epub 2018/09/28. 10.1186/s13071-018-3118-8 . 30257710 PMC6158824

[40] Miri-Moghaddam E, Bameri Z, Mohamadi M. Duffy blood group genotypes among malaria Plasmodium vivax patients of Baoulch population in Southeastern Iran. Asian Pac J Trop Med. 2014;7(3):206–7. Epub 2014/02/11. 10.1016/S1995-7645(14)60021-3 . 24507640

[41] Monhaty SS, S K, Fotedar R, Lakshminarayana J, Parihar R. Prevalance of Duffy blood groups among the population of the desert region of India. J Rural Trop Pub Health. 2011;10:53–6.

[42] Cavasini CE, de Mattos LC, Couto AA, Couto VS, Gollino Y, Moretti LJ, et al Duffy blood group gene polymorphisms among malaria vivax patients in four areas of the Brazilian Amazon region. Malar J. 2007;6:167 Epub 2007/12/21. 10.1186/1475-2875-6-167 . 18093292 PMC2244634

